# Priming Pharyngeal Motor Cortex by Repeated Paired Associative Stimulation: Implications for Dysphagia Neurorehabilitation

**DOI:** 10.1177/1545968312469837

**Published:** 2013-05

**Authors:** Emilia Michou, Satish Mistry, John Rothwell, Shaheen Hamdy

**Affiliations:** 1 University of Manchester, Manchester, UK; 2 Institute of Neurology, University College London, London, UK

**Keywords:** swallowing, motor cortex, paired associative, neurostimulation repetition

## Abstract

*Background*. Several stimulation parameters can influence the neurophysiological and behavioral effects of paired associative stimulation (PAS), a neurostimulation paradigm that repeatedly pairs a peripheral electrical with a central cortical (transcranial magnetic stimulation [TMS]) stimulus. This also appears to be the case when PAS is applied to the pharyngeal motor cortex (MI), with some variability in excitatory responses, questioning its translation into a useful therapy for patients with brain injury. *Objective*. To investigate whether repeated PAS in both “responders” and “nonresponders” could enhance cortical excitability in pharyngeal MI more robustly. *Methods*. Based on their responses after single PAS, healthy participants were stratified into 2 groups of “responders” and “nonresponders” and underwent 2 periods (60 minutes inter-PAS interval) of active and sham PAS in a randomized order. Neurophysiological measurements with single TMS pulses from pharyngeal motor representation were collected up to 90 minutes after the second PAS period. *Results*. Repeated PAS increased cortical excitability up to 95% at 60 minutes following the second PAS in both the “responders” and “nonresponders.” Moreover, cortical excitability in the “nonresponders” was significantly different after repeated PAS compared with single and sham application (*P* = .02; *z* = −2.2). *Conclusions*. Double dose PAS switched “nonresponders” to “responders.” These results are important for PAS application to dysphagic stroke patients who do not initially respond to a single application.

## Introduction

Dysphagia is a major complication following acute neurological and more chronic neurodegenerative disorders,^1,2^ resulting in increased risk of death, pneumonia, dehydration, and malnutrition. Rehabilitation-based interventions have been proposed, and therapeutic protocols have been formed with the scope of targeting cortical and subcortical brain areas that are recruited during the highly coordinated sensorimotor activity of swallowing. These paradigms are based on knowledge from in vivo animal and human studies with electrophysiological methods,^3^ transcranial magnetic stimulation (TMS), and functional magnetic resonance imaging.^4^

Recently, paired associative stimulation (PAS) paradigms have been developed and investigated on both neurophysiological and behavioral measures of swallowing performance.^5^ In this paradigm, pairing a peripheral (pharyngeal electrical stimulation, PES) with a central cortical (TMS) stimulus to the pharyngeal motor cortex (MI) resulted in an increase of cortical excitability of corticomotor projections to the pharyngeal muscles,^5,6^ followed by beneficial behavioral changes. Moreover, following a single application of PAS to the unaffected hemisphere of chronic stroke patients, cortical excitability was increased bilaterally. This was accompanied also by significant functional changes in swallowing physiology and a reduction in the incidence of penetration and/or aspiration of material into the trachea.^5^

However, the effective application of PAS to a dysphagic stroke population may be confounded by several other parameters such as the heterogeneity in responses because of different lesion loci and volumes as well as genetic factors proposed to be influential in the responsiveness to neurostimulation.^7^ Other potential reported parameters for differences in responses have been the inherent intrinsic neuronal activity,^8,9^ time of day for the intervention delivery,^10^ attentional state,^11^ and cortical thickness in primary sensorimotor cortex.^12^

This variability in responsiveness has been observed in the literature for the limbs of both in healthy participants^13^ and in stroke patients.^14^ In the latter patient population study^14^ with 9 hemiparetic stroke patients, functional improvements in motor performance were found, while the group’s variability in responses did not allow for significant changes in neurophysiological measurements. Although this has not been the case for our studies in dysphagic stroke patients,^5^ further investigation into other parameters of this neurostimulation paradigm is imperative, since such knowledge will guide us to address the robustness of PAS for swallowing rehabilitation. In point of fact, one controlled study in 5 healthy subjects showed that lithium (a mainstream medication for bipolar disorders) reversed the cortical excitability of “nonresponders” after excitatory PAS into that similar to “responders.”^15^

Therefore, we investigated the variability in the excitatory responses following the application of the neurorehabilitation paradigm, PAS, in healthy volunteers. Following this initial study, we investigated whether repetitive dosing of PAS could address the variability in responses at the neurophysiological level.

## Participants and Methods

No major illnesses were reported by the 18 healthy participants (4 men; age, 39 ± 3 years [mean ± SEM]; 16 right-handed). Written informed consent was obtained from all participants before the experiments. General practitioners were informed of the participants’ consent prior to the studies. Exclusion criteria included a history of epilepsy; cardiac pacemaker; previous brain or ear, nose, and throat surgery; any history of swallowing problems; significant medical disorders; pregnancy; metal in the head or eyes; or use of medication that acts on the central nervous system. Research protocols were approved by Salford and Trafford Research Ethics Committee, and experiments were undertaken in the clinical laboratories of the Inflammation Sciences Research Group at Salford Royal NHS, UK, in accordance with the Code of Ethics of the World Medical Association (Declaration of Helsinki).

## Experimental Procedures

### Transcranial Magnetic Stimulation

Focal TMS was performed using a flat figure-of-8-shaped magnetic coil (outer diameter 70 mm) connected with a Magstim BiStim^2^ magnetic stimulator (Magstim Co, Whitland, Wales, UK), which produced maximal output of 2.2 T.

### Pharyngeal and Thenar Electromyographic Measurements

Pharyngeal electromyographic measurements after single TMS pulses, termed pharyngeal motor evoked potentials (PMEPs), were recorded through a 3.2-mm diameter intraluminal catheter (Gaeltec Ltd, Isle of Skye, Scotland) with a built-in pair of bipolar platinum ring electrodes, which was inserted either nasally (15-17 cm to pair electromyographic electrodes from the nasal flare) or orally (13-15 cm) depending on subject’s preference. This allowed the recording of PMEPs at the mid-pharyngeal level (middle pharyngeal constrictors).

As a control (unilaterally innervated) system, thenar motor evoked potentials (TMEPs) from the abductor pollicis brevis muscle were also recorded from MI (see supplementary material).

### Paired Associative Stimulation

Paired associative stimulation was delivered by pairing a pharyngeal electrical stimulus (0.2-ms pulse) with a single TMS pulse on the pharyngeal MI at the intensity of resting motor threshold (rMT) plus 20% of magnetic stimulator output (MSO). The 2 paired pulses were delivered in a controlled manner through Signal software (v4.1, Cambridge Electronic Design, Cambridge, UK), with an interstimulus interval of 100 milliseconds, based on previous investigations.^5,6^ The intraluminal catheter used for PMEPs was connected to a constant current generator (model DS7; Digitimer, Herts, UK) to deliver pharyngeal electrical stimulation (PES). The paired pulses were delivered every 20 seconds for a total of 10 minutes, giving 30 paired pulses in total. For the sham intervention, the coil was held tangentially to the skull at a 90° angle to sagittal plane, and no PES was delivered through the catheter in situ (see supplementary material).

## Experimental Protocols

### Protocol 1: Real and Sham PAS on Pharyngeal Corticobulbar Projections

The participants were initially asked to attend the laboratory on 2 occasions. At each attendance, volunteers sat comfortably in a reclining chair with the catheter in situ. The cranial vertex was identified^16^ and marked on the scalp.

The cortical sites for pharyngeal response, characterized as the sites evoking the largest pharyngeal responses in each hemisphere, were identified with mapping procedures using single TMS pulses delivered over multiple points at 80% MSO intensity, as previously described.^5^ The “stronger” pharyngeal projection was defined as the hemispheric site with the lowest rMT to evoke PMEPs, whereas the site with the highest rMT was termed “weaker” pharyngeal projection (see supplementary material).

To assess the effects of real and sham PAS, all participants were studied at least 1 week apart and received 10 minutes of PAS (PAS_10min_) or sham (PAS_Sham_) in a randomized manner using block randomization (StatsDirect Ltd, Cheshire, UK). Measurements of cortical excitability for each hemispheric site (10 pulses at rMT + 20% MSO at stronger pharyngeal, weaker pharyngeal, and thenar representation) were made at baseline and at each of the postintervention follow-up time points (immediately, 30, 60, and 90 minutes) on each visit. During these periods, participants were advised to withhold from any swallowing, coughing, talking, or moving their hands or arms. The lead researcher performed the recordings and the analysis but was blinded to the interventions, delivered by a separate researcher who was blinded to the analysis. Participants’ data were kept unidentifiable.

### Protocol 2: Effects of Repeated PAS Over Pharyngeal MI in Responders and Nonresponders

Following the completion of protocol 1, 12 participants from that protocol were recruited and stratified into 2 groups based on their responses to stimulation of the “stronger” projection (area under the curve [AUC] analysis for PAS_10min_). Six subjects whose responses were at ≥75th percentile of AUC results after PAS_10min_ to the “stronger” pharyngeal projection were termed “responders,” whereas the 6 subjects with ≤25th percentile AUC were termed “nonresponders.” The procedures for recording PMEPs and TMEPs, randomization and blinding were identical to protocol 1. Both groups of “responders” and “nonresponders” underwent

(a) double dose of PAS_10min_ (1 hour intertreatment interval) (Repeat PAS_10min_),
(b) single dose of PAS_10min_ followed by PAS_Sham_ (PAS_10min_ + PAS_Sham_), and
(c) PAS_Sham_ followed by PAS_Sham_ (PAS_Sham_ + PAS_Sham_)

on 3 occasions. Cortical excitability was assessed for up to 60 minutes before the second dose of PAS and then immediately, 30, 60, and 90 minutes after second PAS_10min_ or PAS_Sham_.

## Data Analysis of Neurophysiological Measurements

Peak-to-peak amplitudes of MEPs evoked by TMS were used as a measure of cortical excitability. The individual MEPs were reviewed with Signal Software (CED, Cambridge, UK), MEPs averages were calculated for each time point, and an average trace was created (for response latencies measurements). Baseline MEP data and response latencies for all interventions were compared with nonparametric tests (Friedman test and Wilcoxon signed rank test). Data were normalized to baseline and are shown as percentage change from baseline to minimize interindividual variability. Interindividual factors such as age and sex were therefore equalized. Changes in excitability over time were compared (excluding baseline) using a generalized linear model repeated-measures analysis of variance (rmANOVA; SPSS 16.0). In addition, AUC from percentage change analysis was employed to show the integrated magnitude of the responses of the participants, thus eliminating time-dependency effects. A *P* < .05 was taken as a measure of statistical significance. All data are presented as group mean ± SEM, unless stated otherwise.

## Results

### Protocol 1: Real and Sham PAS on Pharyngeal Corticobulbar Projections

PMEPs were recorded in all subjects without any adverse incidents. Larger pharyngeal responses were found from the right hemisphere in 5 participants, whereas the remaining subjects had larger responses from the left hemisphere. The optimal site for stimulation anterior to the vertex was located at 4.6 ± 0.2 cm for the right and 4.9 ± 0.2 cm for the left hemisphere and lateral to midline was 3.8 ± 0.6 cm (right) and 3.7 ± 0.6 cm (left). The mean value of pharyngeal rMT for the “stronger” pharyngeal projection, where PAS was applied, was 67% ± 3% MSO. PES as part of PAS was delivered at 16.6 ± 3.5 mA.

#### Baseline TMS response amplitudes and latencies

Baseline cortical excitability for the 2 different studies remained stable for pharyngeal and thenar projections (see supplementary material).

#### Changes in cortical excitability

A 3-way rmANOVA on percentage change after PAS_10min_ and PAS_Sham_ with factors of *Intervention, Time*, and *Site* (strong pharyngeal, weak pharyngeal, thenar projection) revealed a significant *Intervention* × *Time* × *Site* interaction (*F*_1,17_ = 6.83; *P* = .018) and was further analyzed below.

#### Changes in PMEP-strong

A 2-way rmANOVA on the percentage change with the factors: *Intervention* (PAS_10min_, PAS_Sham_) and *Time* revealed significant *Time* × *Intervention* interaction (*F*_1,17_ = 6.37; *P* = .022) and a significant effect of intervention for PAS_10min_ against PAS_Sham_ (*F*_1,17_ = 16.22; *P* = .001). Compared with PAS_Sham_, PAS_10min_ increased cortical excitability (maximum of 62% ± 23%, 60 minutes). PMEP amplitudes increased significantly immediately (*P* = .012; 95% confidence interval [CI] −87.02 to −12.3) and at 30 minutes (*P* = .01; 95% CI = −52.8 to −7.04) after PAS_10min_ compared with baseline. Cortical excitability was still significantly increased up to 51% ± 20% (*P* = .04; 95% CI = −72 to −0.06) at 90 minutes.

#### Changes in PMEP-weak

For the “weaker” (nonstimulated) pharyngeal projection, a significant *Time* × *Intervention* interaction was observed (*F*_1,17_ = 6.6; *P* = .02), but there were no significant effects of *Intervention* or *Time*.

#### Changes in TMEP (control)

TMEP response amplitudes and latencies following PAS_10min_ and PAS_Sham_ were unaffected (see supplementary material).

#### AUCs—strong and weak

Nonparametric statistical test (Friedman test) on AUCs of percentage change after PAS_10min_ and PAS_Sham_ showed significant differences in distribution (*P* < .001; χ^2^ = 19.6). Wilcoxon tests performed on AUC after PAS_10min_ and PAS_Sham_ showed significant difference only for the “stronger” pharyngeal projection compared with PAS_Sham_ (*z* = −3.33; *P* = .001), verifying the aforementioned results (see Table 1 and Supplementary material). The subjects were then stratified to “responders” (≥75th percentile) and “nonresponders” (≤25th percentile).

**Table 1.** Group Changes in Cortical Excitability After Real and Sham PAS^a^

	Strong Projection				Weak Projection		
	25th Percentile	Median	75th Percentile		25th Percentile	Median	75th Percentile
PAS_10min_	15.67	148.9	235.9	§	−68.9	0.89	85.5
PAS_Sham_	−104.2	−55.9	−27.0		−109.8	−40.5	8.22

Abbreviations: PAS, paired associative stimulation; PMEP, pharyngeal motor evoked potential.

a Group mean area under the curve (calculated by the percentage change in PMEPs’ amplitude against time) analysis of “stronger” and “weaker” pharyngeal projection after PAS_10min_ and PAS_Sham_. There was a significant difference between the change in cortical excitability of the “stronger” projection following real PAS compared with sham (^§^, *z* = −3.33, *P* = .001).

### Protocol 2: Effects of Repeated PAS Over Pharyngeal MI in Responders and Nonresponders

**Figure 1.**
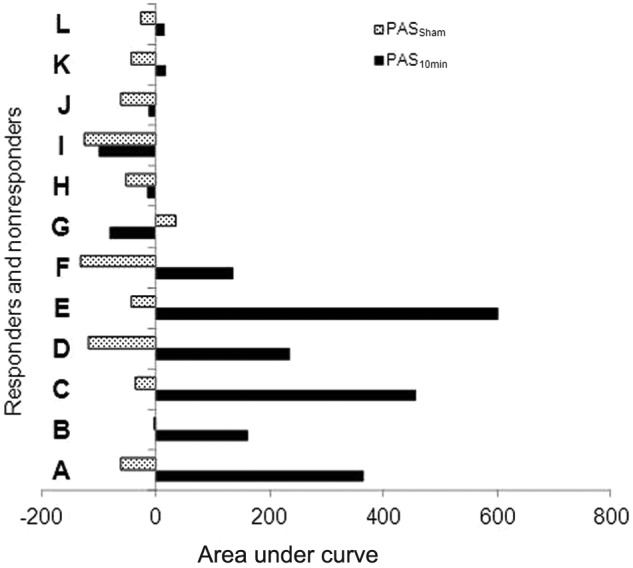
Area under the curve results of the individual subjects participating in protocol 2. The 12 subjects shown above were selected according to their responses after the completion of protocol 1. Subjects A to F were termed as ‘responders,’ since they showed an increase in AUC of cortical excitability after single PAS. Subjects G to L were termed as ‘non-responders,’ based on the minimal changes observed after single real PAS_10min_. Abbreviations: AUC, area under the curve; PAS, paired associative stimulation.

Twelve volunteers (11 women; age, 43 ± 8 years, mean ± SEM) were invited to participate in protocol 2, and Figure 1 shows their responses for PAS_10min_ on the “stronger” pharyngeal projection. As with protocol 1, all measurements in protocol 2 were recorded with no adverse incidents (see supplementary material).

#### Cortical excitability changes for “responders” and “nonresponders”

Baseline TMS response amplitudes were similar and latencies remained unaffected across the 3 arms for all sites (see supplementary material).

#### Changes in cortical excitability

A 3-way rmANOVA with factors *Intervention* (Repeat PAS_10min_, PAS_10min_ + PAS_Sham_, PAS_Sham_ + PAS_Sham_), *Time* (immediately, 30, 60, and 70 minutes; 90, 120, and 150 minutes) and *Site* (strong, weak, and thenar projection) showed a significant interaction of *Intervention* × *Time* × *Site* interaction (*F*_1,11_ = 8.60; *P* = .016). Three separate 2-ways rmANOVAs were performed for each hemispheric *Site* with factors *Intervention* and *Time*.

#### Changes in PMEP-strong

**Figure 2.**
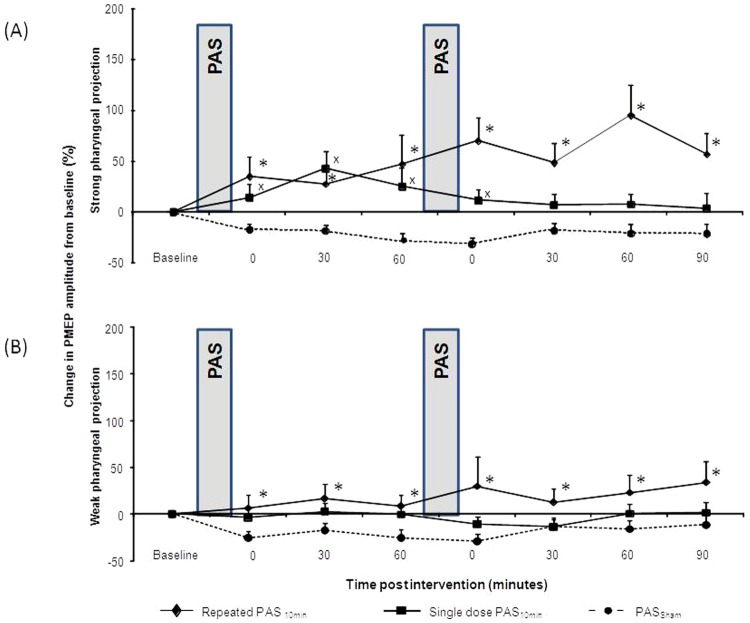
Group mean percentage change in PMEPs amplitude, on the ‘stronger’ (stimulated) (A) and ‘weaker’ (B) pharyngeal projection following different PAS doses and sham stimulation. Increase in amplitude in the ‘stronger’ pharyngeal projection was observed following both repeated PAS_10min_ (♦) (*P=.001) and single PAS_10min_ (■) for the initial period after the first application up to 60 minutes (^x^P=.012), compared to sham PAS_10min_ (●). For the ‘weaker’ pharyngeal projection (B) only repeated PAS10min resulted in significant increase in cortical excitability (*P=.025). Abbreviations: PMEP, pharyngeal motor evoked potential; PAS, paired associative stimulation.

Figure 2A shows the changes in cortical excitability of the stimulated-“stronger” pharyngeal projection in all subjects for the 3 different studies. There was a significant *Time* × *Intervention* interaction (*F*_1,11_ = 20.4; *P* = .001). Moreover, there was a significant difference between the interventions of repeated PAS_10min_ versus PAS_Sham_ (*F*_1,11_ = 19.5; *P* = .001) and a significant difference between repeated PAS_10min_ versus PAS_10min_ + PAS_Sham_ (*F*_1,11_ = 8.7; *P* = .013). The effect of the application of single PAS_10min_ was also significantly different compared with PAS_Sham_ (*F*_1,11_ = 9.15; *P* = .012).

The maximum increase in group percentage change (up to 95% ± 29%) was observed 60 minutes after repeated PAS_10min_, whereas after the single application of PAS_10min_, maximum percentage change reached 25% ± 18% at 60 minutes (Figure 2A).

#### Changes in PMEP-weak

There was a significant interaction of *Time* × *Intervention* for the “weaker” (nonstimulated) pharyngeal site (*F*_1,11_ = 6.2; *P* = .029). However, only the effects of repeated PAS_10min_ were significantly different compared with PAS_Sham_ (*F*_1,11_ = 6.6; *P* = .025) with excitability maximally increasing to 34% ± 22% at 90 minutes, whereas after the single PAS_10min_, this was 2.6% ± 9% at 30 minutes after the initial PAS_10min_ (Figure 2B).

#### Changes in TMEP

Application of repeated and single PAS_10min_ did not change thenar muscle excitability as compared with sham (see supplementary material).

#### Responders versus nonresponders

**Figure 3.**
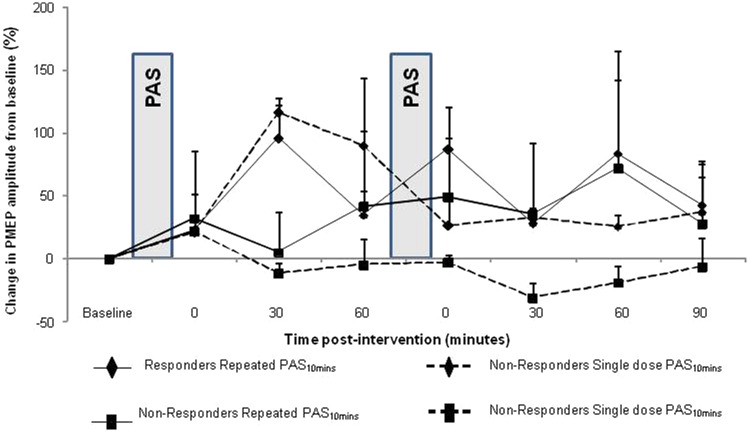
Group mean percentage change in PMEPs amplitude on the ‘stronger’ (stimulated) pharyngeal motor representation following repeated (■) and single (♦) PAS in ‘responders’ (solid line) and ‘non-responders’ (dashed line). Both ‘responders’ and ‘non-responders’ presented the same within group change in excitability after the initial PAS across the two study arms. Differences in responses are observed for both groups after the application of repeated PAS_10min_, here compared to the responses after single application of PAS_10min_ for each group.

The percentage changes for “responders” and “nonresponders” after repeated and single PAS are shown in Figure 3.

Two different AUC analyses were performed and compared with nonparametric tests. We first included each group’s responses for all time points up to the end point (method A). We then calculated the effects of each application (PAS_10min_ or PAS_Sham_) up to 60 minutes following baseline and up to 90 minutes following the second application of PAS_10min_ or PAS_Sham_ (method B).

#### Method A

Friedman χ^2^ was 39.7 and gave *P* values of <.001 (stronger projection) and 10.3 with *P* = .006 (weaker projection), suggesting that the distributions were different for both pharyngeal projections. Nonparametric tests were then performed to capture the differences between “responders” and “nonresponders.” Table 2 presents the different responses of the group of “responders” and “nonresponders” for both projections across all interventions.

**Table 2.** Area Under the Curve of Percentage Change in the Amplitude on the “Stronger” (Stimulated) and “Weaker” Pharyngeal Projection Following Repeated, Single, and Sham PAS in “Responders” and “Nonresponders”^a^

		Strong Projection						Weak Projection					
		Responders			Nonresponders			Responders			Nonresponders		
		25th Percentile	Median	75th Percentile	25th Percentile	Median	75th Percentile	25th Percentile	Median	75th Percentile	25th Percentile	Median	75th Percentile
Repeat PAS_10min_	Real	75.6	172.4	289	−134.1	−45.3	18.0	−27.9	40	121.7	−48.5	−11.2	53.8
	Real	134.2	31.1	631.1	57.9	111	148.1	−66.4	62.2	219.4	−64.6	−3.8	208
Single PAS_10min_	Real	91	154.1	203.7	−53.9	−35.3	8.5	−67.1	−31.4	30.9	−31	8.1	142.4
	Sham	6	130.7	211	−16.8	−54.1	−12	−131.9	−78.8	9.3	−23.1	26.5	119
Sham PAS	Sham	−78.7	−33.8	−13.1	−87.2	−45.4	−18.7	−118	−57.1	−9.9	−116	−32.1	2.77
	Sham	−126.2	−61.8	−33.7	−184.2	−62.3	−15.4	−139.5	−87.9	1.2	−178.9	−15.1	32.3

a The connecting lines present the pairs with significant difference (nonparametric Wilcoxon tests, *P* < .05), showing that the repeat of PAS_10min_ for both “responders” and “nonresponders” resulted in further increase in cortical excitability in “stronger” pharyngeal projection. For the “weaker” pharyngeal projection, the repeated PAS_10min_ resulted in a further increase, significant only compared with PAS_Sham_.

#### Method B

Repeated application of PAS_10min_ further increased the excitability for responders compared with the initial application for the “stronger” projection (*z* = −2.2; *P* = .02). The effects of both initial and repeat PAS_10min_ were significantly different compared with PAS_Sham_ both for the stronger and weaker projections for “responders” (both: *z* = −2.2; *P* = .02). Importantly, responders’ AUCs after repeated PAS were also increased compared with single PAS_10min_ for the same period (*z* = 1.94; *P* = .046). As expected, the effects of single PAS_10min_ were significantly different compared with sham for both the initial 60 minutes and for up to 150 minutes for the “responders,” indicative that in “responders” a single application of PAS may induce long-term effects.

Repeated PAS_10min_ also resulted in an increase to the stronger pharyngeal projection in “nonresponders” (*z* = −2.2; *P* = .02), which was significantly different when compared with single PAS and PAS_Sham_ (*z* = −2.2; *P* = .02). There was no difference between the effects of the single active PAS and the effects after PAS_Sham_ or the effects of the first period of stimulation in the double dose PAS arm for the “nonresponders,” in keeping with previous results for the reduced effects following single PAS to “nonresponders” in protocol 1.

## Discussion

The effects of PAS_10min_ on the “stronger” pharyngeal projection corroborate the results of our previously published data^5^ and show that PAS has the potential to excite the swallowing neural network. Most important, this study set out to examine the effects of repeated PAS_10min_ in 2 groups of subjects in whom PAS was either excitatory or ineffective and to investigate whether PAS repetition could further modulate MI excitability. Our observation that additional doses of PAS have the potential to convert “nonresponders” to “responders” is of interest and merits further discussion.

## Dose Effects of PAS on Bilateral Pharyngeal MI

Repeated PAS_10min_ over the “stronger” pharyngeal projection in both “responders” and “nonresponders” induced facilitation in both stimulated and unstimulated hemispheres, with cortical excitability in the stimulated MI being significantly increased after the second application. The magnitude of these facilitatory effects is surprising, since the group consisted of equal numbers of “responders” and “nonresponders.” Separate analysis for the effects of repeated PAS to “responders” and “nonresponders” individually (controlled with single and sham PAS) indicated that the effects were mainly because of the second PAS.

Previous work on limb muscles in healthy volunteers,^17^ stroke patients,^14^ and animal models^18^ have shown that repeated PAS once per day for 5 days or even longer (ie, in stroke patients)^14^ enhanced neurophysiological properties of the corticomotor system, as measured by MEP amplitude, accompanied with behavioral benefits.^14,18^ However, results from our studies are not directly comparable, since the repeat PAS protocol was applied within a shorter epoch to the initial intervention.

Notwithstanding, the findings from our current study differ from those by Müller et al.^8^ These authors found that when cortical excitability was conditioned with a PAS paradigm that enhances long-term potentiation (LTP), then the application of a second LTP-like PAS intervention resulted in cortical depression. The results from that study fall within the well-described theory of Bienenstock–Cooper–Munro,^19^ which tries to elucidate the way that neuronal systems reach homeostasis and balance inhibitory and facilitatory interactions over a period of time, originally observed in visual cortical neurons. In contrast, our data have shown that the effect of the first PAS_10min_ application resulting in LTP-like plasticity in pharyngeal motor cortex was further enhanced after a second facilitatory PAS_10min_ application. This finding requires further consideration.

There are likely to be a number of explanations for the difference in the results in the swallowing model. First, the existence of the “ceiling effect,” the extent to which cortical excitability can be further increased, has not been examined for the swallowing motor system. Second, the effect of “saturation” of the cortical capacity for synaptic efficacy and LTP^20^ has also not been investigated in detail for swallowing. However, previous PAS studies showed that 30 minutes of stimulation did not produce significant changes compared with shorter durations.^5^ The inter-PAS interval is also an important parameter to consider for the modulatory effects of PAS to MI. In the study by Müller et al,^8^ the inter-PAS interval was 30 minutes, shorter than the 60-minute inter-PAS interval in our protocol. Furthermore, we have previously shown that the effects of single PAS targeting pharyngeal MI can last up to 90 minutes.^5,6^ In this context, evidence from the use of transcranial direct current stimulation in the limb MI has shown that when the repeated application falls within the excitatory window of the initial input, the after effects are increased.^21^ In addition, the interval between the pairs of peripheral and cortical stimulation is critically important: Literature by others^22^ has suggested that with different intervals between pairs, different mechanisms contribute to the effects of PAS.

Moreover, at this stage of research, it is still unclear as to whether the changes in cortical excitability are because of changes in the efficacy of the synaptic connections or changes in neuronal excitability. Cortical and subcortical brain areas are activated in an interconnected network during swallowing. Whether the change in cortical excitability following the first application of PAS would spread to connected brain regions of the swallowing network resulting in a further increase after the application of repeat PAS is uncertain. Further studies with neuroimaging techniques may help determine this and would be of importance for the applications of neurorehabilitation to the corticobulbar network for swallowing.

More recently, it has been shown that PAS effects in limb MI can be remotely influenced by cerebellar stimulation. Modulation of cerebellar activity using transcranial direct current stimulation was able to abolish the excitatory effects of PAS in the motor cortex.^22^ These findings suggest that combining neurostimulation inputs is both modality and region specific, which supports the contention that pharyngeal motor cortex neurostimulation might behave differently in other regions, when PAS is applied.

Nonetheless, our study also raises the possibility that delivering initial PAS as a form of conditioning changed the threshold for synapses to engage in “nonresponders” (producing an “imbalance” in activity), and the repeat PAS has enabled these “activated” synapses to be strengthened more easily. However, in vivo studies to validate this assumption are difficult to perform, given that the measurement of excitability is not directly equal to synaptic activity.^23^ Nevertheless, such suggestion could hold considerate value for the rehabilitation of swallowing disorders, if we take into consideration that LTP induced by targeted PAS, such as in our study, has similarities with LTP resulting from motor training and learning,^24^ the latter being important in the case of dysphagia rehabilitation.^3^

In conclusion, we report evidence that subjects who do not respond to an initial application of excitatory stimulation (PAS_10min_) can show an increase in MEP responses after a repeated excitatory PAS_10min_; these effects being larger than when compared with a single application. This has implication for PAS application to dysphagic stroke patients who may not respond to single doses of stimulation and provides the example for other neuromodulatory interventions under investigation for customized approaches when applied to the swallowing neural network. Future utilization of the repeated approach in stroke patients with dysphagia and neuroimaging studies seem warranted, since PAS appears to hold promise as a powerful neurorehabilitation paradigm for dysphagia rehabilitation after stroke. Double PAS could therefore drive cortical plasticity during the critical period of plasticity in the weeks following a stroke and may substantially enhance traditional therapy.^25^
